# Analysis of the efficacy of multidisciplinary integration based on 3D reconstruction technology for the treatment of gout stone

**DOI:** 10.1186/s13018-025-05506-8

**Published:** 2025-02-04

**Authors:** Shizhe Zhou, Zengxiao Zhang, Tian Liu, Yijun Xu, Yuehai Pan, Ying Chen

**Affiliations:** 1 Department of Endocrinology and Metabolism, The Affiliated Hospital of Qingdao University, Qingdao, China; 2 Department of Endocrinology and Metabolism, Beijing Tsinghua Changgung Hospital, School of Clinical Medicine, Tsinghua University, Beijing, China; 3 Department of Otorhinolaryngology Head and Neck Surgery, The Affiliated Hospital of Qingdao University, Qingdao, China; 4 Department of Hand and Foot Surgery, The Affiliated Hospital of Qingdao University, Qingdao, China; 5 The Affiliated Hospital of Qingdao University, No. 59, H16 Jiangsu Road, Shinan District, Qingdao, Shandong 266003 China

**Keywords:** Gout stone, 3D reconstruction, Surgical, Uric acid, SF-36

## Abstract

**Objective:**

This study investigates the efficacy of multidisciplinary fusion therapy based on 3D reconstruction technology for the treatment of gouty stone by comparing the efficacy of multidisciplinary fusion therapy with pharmacologic therapy.

**Methods:**

This study is a cohort study.Patients who underwent gout stone surgery at the Affiliated Hospital of Qingdao University from November 2020 to November 2022 were included in this study, totaling 85 to form the MDT surgery group, and matched among gout stone patients in the outpatient clinic during the same period to form the medication group. Patients in the 2 groups were followed up for 6 months to compare baseline and follow-up data.

**Results:**

Both groups experienced a decrease in uric acid levels and an increase in SF-36 scores during follow-up. After adjusting for confounders, multifactorial logistic regression showed that the uric acid attainment rate of patients in the MDT surgery group was 4.011 times higher than that of the drug group (OR: 4.011, 95% CI: 1.595, 10.086, *P* = 0.003); the proportion of patients with an increase in SF-36 in the MDT surgery group was 4.976 times higher than that of the drug group (OR: 4.976, 95% CI: 2.243, 11.040, *P* < 0.001); the proportion of patients treated with high-dose medication in the MDT surgery group was 1.8% of that of patients in the drug group (OR: 0.018, 95% CI: 0.002, 0.148, *P* < 0.001); and the proportion of patients in the MDT surgery group who developed frequent gout was 2.8% of that in the drug group (OR: 0.028 95% CI: 0.003, 0.2398, *P* = 0.001). the proportion of patients in the MDT surgery group who developed abnormal liver function was 0.317 times higher than that in the drug group (OR: 0.317, 95% CI: 0.121, 0.831, *P* = 0.019).

**Conclusion:**

The multidisciplinary integration of 3D reconstructive techniques for gout stone treatment resulted in an increase in uric acid compliance, a decrease in the frequency of gout and the appearance of liver impairment; and a greater benefit in terms of improvement in the quality of life of the patients after treatment.

**Supplementary Information:**

The online version contains supplementary material available at 10.1186/s13018-025-05506-8.

## Introduction

Disorders of purine metabolism lead to elevated blood uric acid concentration, formation and deposition of monosodium urate (MSU) crystals locally in the joints, inducing a localized inflammatory response and tissue destruction, known as gout [1, 2]. The main feature of gout is the gout stone, which is a complex mass composed of MSU crystals, multiple immune and inflammatory cells, and a fibrous capsule [3, 4]. Gout stones complicate approximately 12-35% of gout patients, usually appearing more than a decade after the 1st gout attack, and can lead to a significant reduction in the patient’s quality of life [5]. Some studies have shown that gout stones can cause wasting muscle atrophy [6, 7]; in severe cases, they can be teratogenic and disabling. In addition, the presence of gout stones in gout patients has been associated with an elevated risk of death [8–10].

The latest American College of Rheumatology guidelines for the treatment of gout state that “serum urate should be reduced to less than 5 mg/dl in patients with gouty stones“ [11]. Although there appears to be a significant correlation between the degree of serum uric acid reduction and the resolution of gouty stones [12–14], and oral drug uric acid-lowering therapies are able to reduce serum uric acid by 50–60% to levels between 4 and 6 mg/dl [15], this may not be sufficient to rapidly resolve gouty stones. genetic mutations, unhealthy lifestyles, poor medication compliance, and comorbidities can potentially make it difficult to achieve the desired uric acid control goal, which is insufficient to rapidly abate gout stones. Surgical treatment is considered when medications are poorly controlled, when gout stones develop ulcers, infections, or severe joint damage and pain. Although surgical treatment of gout stones can remove local gout crystals and reduce the total amount of uric acid in the body, it is not effective in reducing the recurrence rate of gout, let al.one curing gout; local soft tissues may be damaged during surgery, and substandard control of uric acid in the postoperative period increases the probability of secondary surgery. Patients with severe gouty stone have limited recovery of joint function after surgery, and may even experience postoperative complications such as poor wound healing or infection. Three-dimensional CT can make gouty stone lesions appear green, three-dimensional precise distinction from normal bone, more accurate localization of deeper gouty stone, and reduce the damage to local soft tissue [16, 17].

The treatment of gouty stone is a long-term process, the diagnosis and treatment involves multiple disciplines, and the process of patients seeking medical treatment in different departments is more complicated, which leads to decreased compliance. For this reason, we have developed a multidisciplinary integration of gouty stone treatment based on 3D reconstruction technology, and the aim of this study is to investigate the efficacy of multidisciplinary integration of 3D reconstruction technology on gouty stone patients, with a view to discovering a more reasonable and reliable treatment plan for gouty stone patients.

## Method

### Subject of the study

**Fig. 1.1:**
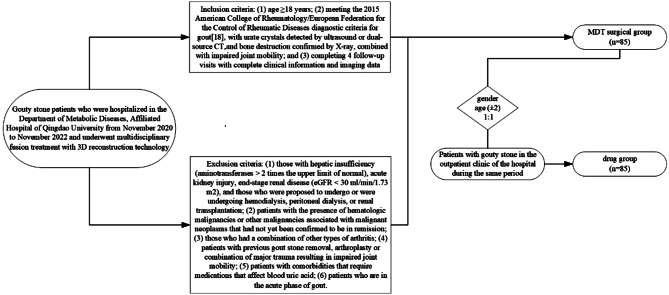
Flowchart of enrollment of study subjects in the MDT surgery and drug groups

This is a retrospective cohort study. Gouty stone patients who were hospitalized in the Department of Metabolic Diseases, Affiliated Hospital of Qingdao University from November 2020 to November 2022 and underwent multidisciplinary fusion treatment with 3D reconstruction technology were included in this study. Inclusion criteria: (1) age ≥ 18 years; (2) meeting the 2015 American College of Rheumatology/European Federation for the Control of Rheumatic Diseases diagnostic criteria for gout [18], with urate crystals detected by ultrasound or dual-source CT, and bone destruction confirmed by X-ray, combined with impaired joint mobility; and (3) completing 4 follow-up visits with complete clinical information and imaging data. Exclusion criteria: (1) those with hepatic insufficiency (aminotransferases > 2 times the upper limit of normal), acute kidney injury, end-stage renal disease (eGFR < 30 ml/min/1.73 m2), and those who were proposed to undergo or were undergoing hemodialysis, peritoneal dialysis, or renal transplantation; (2) patients with the presence of hematologic malignancies or other malignancies associated with malignant neoplasms that had not yet been confirmed to be in remission; (3) those who had a combination of other types of arthritis; (4) patients with previous gout stone removal, arthroplasty or combination of major trauma resulting in impaired joint mobility; (5) patients with comorbidities that require medications that affect blood uric acid; (6) patients who are in the acute phase of gout. Dropout/exclusion criteria: (1) those who did not follow the doctor’s instructions for treatment and rehabilitation activities; (2) those who did not follow up the appointment or missed the visit. The study was approved by the Hospital Ethics Committee of the Affiliated Hospital of Qingdao University (approval document: QYFY WZLL 27652), and informed consent was obtained from the patients and their families, who signed an informed consent form. A total of 85 study subjects were included to constitute the MDT surgical group. Patients with gouty stone in the outpatient clinic of the hospital during the same period were matched to the drug group according to gender and age (± 2) 1:1, with the same nativity criteria as in the MDT surgery group. The flowchart of enrollment of study subjects, is shown in Fig. 1.1.

### Research methodology

#### Combined multidisciplinary treatment model

**Fig. 1.2:**
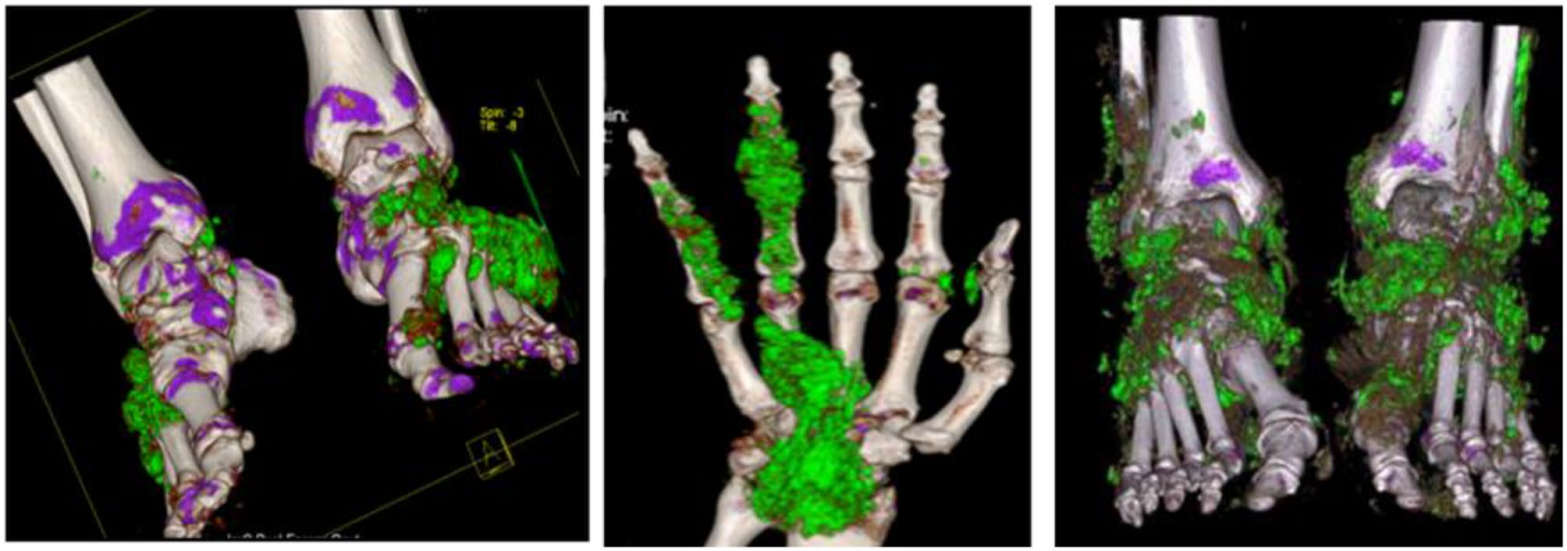
Preoperative three-dimensional CT image

The patient was treated and cared for in the Department of Metabolic Diseases throughout the perioperative period, and the hand and foot surgeons were responsible for performing the gout stone lesion resection. (1) Preoperatively: the metabolic disease department doctors evaluated the patient and performed CT three-dimensional reconstruction of the gout stone site (see Fig. 1.2); they formulated a targeted low purine diet program, analgesic program, and gave treatments such as lowering uric acid and alkalinizing urine. (2) Intraoperative: hand and foot surgeons refer to the gout stone after three-dimensional CT reconstruction images in the gout lesion excision of gout stone tissue, with a large number of 5% sodium bicarbonate injection to flush the wound, intermittent suture wounds, wounds aseptically bandaged, and leave drainage, as appropriate. (3) Postoperative: individualized analgesic program and rehabilitation exercise program were formulated; suture removal was given after 14 days postoperatively depending on the healing of the incision; treatment such as lowering uric acid and anti-infection was given. After discharge from the hospital, rehabilitation exercise, medication guidance, gout home care mission and outpatient follow-up reminders.

#### Medication

The patient was seen in the outpatient clinic of the Department of Metabolic Diseases and was initially given 20 mg of febuxostat for uric acid lowering, with guidance on a low purine diet and exercise regimen during the follow-up period. At the follow-up node, febuxostat was increased by 20 mg according to the patient’s uric acid level.

#### Study protocol

Baseline information was collected 1 day before surgery or at the initial outpatient visit: (1) general information: gender, age, past history, smoking history, drinking history, family history, and history of gout; height, weight, and blood pressure were measured, and the body mass index was calculated as weight (kg)/[height (m)] ^2^. The patient was asked to take a break of ≥ 5 min before the blood pressure measurement, and was asked to be relaxed during the measurement. Blood pressure was measured twice with an interval of 1 min each time, and the average of the two measurements was taken as the final blood pressure value. (2) Biochemical indexes: Fasting blood was collected early in the morning after at least 8 h of dietary abstinence, and was tested for alanine aminotransferase (ALT), azelaic aminotransferase (AST), glucose (GLU), triglyceride (TG), total cholesterol (TC), urea nitrogen (BUN), creatinine (Crea), and uric acid (UA) by the fully automated biochemical method. Follow-up visits were performed at 2 weeks (2 W), 1 month (1 M), 3 months (3 M), and 6 months (6 M) after surgical treatment or application of medications, and treatment was followed as prescribed by the physician during the follow-up period. Investigators recorded information on the frequency, date, duration and severity of gout attacks; SF-36; uric acid and transaminase levels at each follow-up visit since the last visit or follow-up. They were measured using a fully automated biochemical analyzer (Cobas8000; Roche Diagnostics Ltd., Basel, Switzerland).

### Diagnostic criteria and definitions

Gout was diagnosed with reference to the guidelines developed by the ACR/European League Against Rheumatism (EULAR) in 2015 [18]; imaging suggestive of the presence of monosodium urate crystals was used as a diagnostic criterion for gouty stones. The Medical Outcomes Research Short Form-36 (SF-36) consists of eight scaled scores that are weighted sums of the questions in its sections. Assuming that each question is equally weighted, each scale is directly converted to a 0-100 scale. The lower the score, the greater the disability, and the higher the score, the better the health. Uric acid attainment: uric acid < 300 µmol/l defined as 1; medication dose: febuxostat > 40 mg defined as 1; frequency of gouty attacks: gouty attacks/6 months > 1 defined as 1; and change in SF-36: SF-36 (6 M) - SF-36 (baseline) > 0 defined as 1.

### Sample size calculation

The main observation in this study was blood uric acid, and the sample size was calculated based on the blood uric acid levels of the patients in the pre-experimental MDT surgical and drug groups. The two-sided test level alpha was taken as 0.05 and test efficacy as 0.9, allowing for the presence of a 20% number of dropout cases, which was calculated to require a minimum of 83 patients.

### Statistical analysis

Continuous variables that conformed to normal distribution were statistically described as mean ± standard deviation ($\:\bar{X}$± SD), and independent samples t-test analyzed the difference between the baseline information of the two groups; continuous variables that did not conform to normal distribution were statistically described as median, interquartile spacing (M, IQR), and Mann-Whitney U test analyzed the difference between the baseline information of the two groups. Categorical variables were statistically described as frequency, constitutive ratio (N, %), and chi-square test was used to analyze the difference between the baseline information of the two groups. Changes in baseline and follow-up parameters of the enrolled patients were compared using repeated measures ANOVA. The effect of different treatments on observed parameters was analyzed using multifactorial regression. Logistic regression was used to analyze the risk of different treatments on the development of liver function abnormalities after treatment. The processing of missing values is a combination of multiple interpolation and direct deletion. Differences were considered statistically significant at *P* < 0.05. IBM SPSS Statistics 26.0 software was used for data analysis.

## Results

### Basic information of the study populations in the MDT surgery group and the drug group

**Fig. 2:**
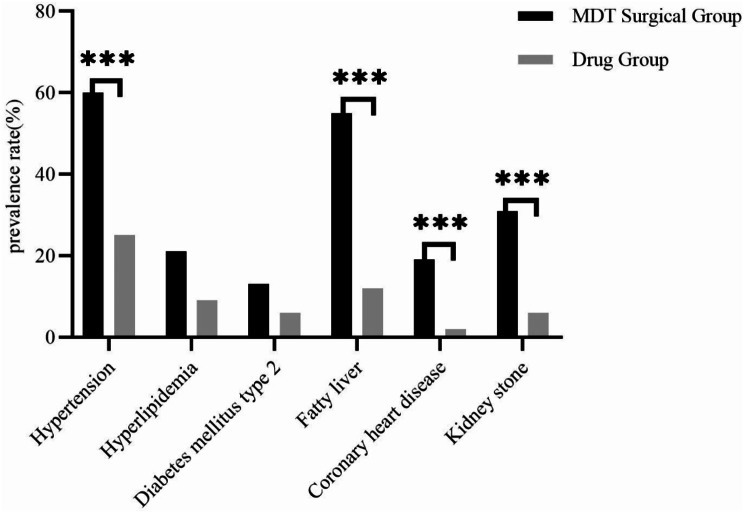
Comparison of co-morbidities between the two groups

A total of 170 patients were included in the study (85 in the MDT surgery group and 85 in the drug group), of which 167 were male, aged 22–85 years, with an average age of 50 years; duration of the disease was 1–36 years, with an average of 12.6 years; site of onset: joints of the hands and feet and subcutaneous multinodular nodules, including the carpal joints with carpal tunnel syndrome. The differences in gender, age, height, weight, BMI, systolic blood pressure, and smoking history between the two groups were not statistically significant (*P* > 0.05); compared with the drug group, the MDT surgery group had lower diastolic blood pressure, longer disease duration, and a smaller proportion of history of alcohol consumption and family history of gout; and the MDT surgery group had higher levels of blood glucose, triglyceride, total cholesterol, and uric acid, and the differences were statistically significant (*P* < 0.05) (See Table 1). The MDT surgery group had a higher proportion of combined hypertension, fatty liver, coronary heart disease, and kidney stones, and the difference was statistically significant (*P* < 0.05) (See Fig. 2).

**Table 1:** Basic information about the study population

Characteristics	MDT Surgical Group(*n* = 85)	Drug group(*n* = 85)	*P*
Males, n(%)	83 (97.65%)	84 (98.82%)	0.560
Age(years)	50.85 ± 6.43	49.39 ± 13.11	0.515
Height(cm)	172.99 ± 6.43	174.06 ± 6.62	0.286
Weight(kg)	81.61 ± 17.10	82.22 ± 16.28	0.814
BMI (kg/m2)	26.85 ± 4.80	27.01 ± 4.25	0.817
SBP(mmHg)	142.75 ± 20.74	144.67 ± 23.86	0.577
DBP(mmHg)	86.40 ± 13.97	91.53 ± 14.58	0.020
Complicating disease			
Hypertension	51 (60.00%)	21 (24.71%)	<0.001
Hyperlipidemia	18 (21.18%)	8 (9.41%)	0.033
Diabetes mellitus type 2	11 (12.94%)	5 (5.88%)	0.115
Fatty liver	47 (55.29%)	10 (11.76%)	<0.001
Coronary heart disease	16 (18.82%)	2 (2.35%)	<0.001
Kidney stone	26 (30.59%)	5 (5.88%)	<0.001
Smoking	40 (47.06%)	49 (57.65%)	0.167
Drinking	48 (56.47%)	63 (74.12%)	0.016
Family history	18 (21.18%)	50 (58.82%)	<0.001
Course of gout(years)	10.00 (11.00)	9.00 (9.00)	0.006
Multiple tophus, n(%)	73 (85.88%)	64 (75.29%)	0.081
Frequent gout attacks, n(%)	78 (91.76%)	75 (88.24%)	0.443
ALT(mmol/l)	23.00 (19.00)	26.00 (22.5)	0.293
AST(mmol/l)	20.00 (10.00)	20.00 (8.00)	0.952
GLU(mmol/l)	5.10 ± 1.42	5.80 ± 0.70	<0.001
TG(mmol/l)	1.56 (0.95)	1.77 (1.48)	0.012
TC(mmol/l)	4.55 ± 0.95	5.30 ± 1.19	<0.001
BUN(mmol/l)	6.10 (3.02)	5.40 (2.95)	0.210
CREA(mmol/l)	80.00 (26.00)	90.00 (19.5)	0.133
SUA(µmol/l)	480.00 (195.00)	563.00 (150.50)	0.001

SBP, systolic blood pressure; DBP, diastolic blood pressure; SUA, serum uric acid; FPG, fasting plasma glucose; TC, total cholesterol; TGs, triglycerides; BUN, blood urea nitrogen; CREA, creatinine; ALT, alanine aminotransferase; AST, aspartate aminotransferase; BMI, body mass index

### Follow-up data analysis of the study population in the MDT surgery group and the drug group

#### Uric acid

**Fig. 3:**
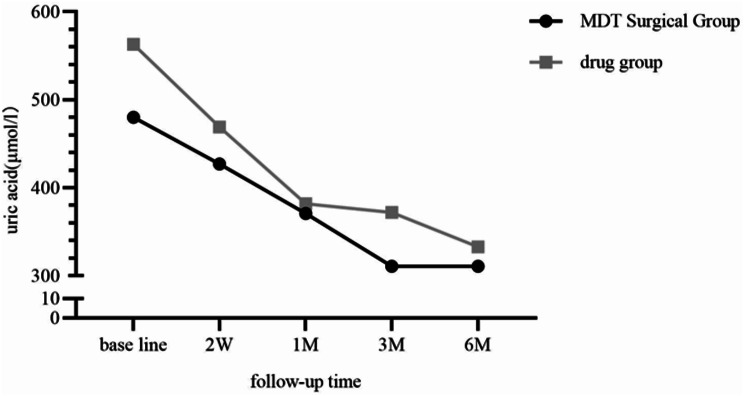
Changes in uric acid over time. (F:126.497, *P*<0.001; F _interaction_:2.720, P _interaction_:0.042; F_intergroup_:12.105, P_intergroup_:0.001)

There was a statistically significant difference in uric acid levels between pre-treatment and multiple follow-ups in the MDT surgery group (F = 61.53, *P* < 0.001), with uric acid levels at baseline > 2 weeks > 1 month > 3 months, with a statistically significant difference (*P* < 0.05) (See Table 2). Uric acid levels at 6 months after MDT surgical treatment were lower than those at 6 months after drug treatment, and the difference was statistically significant; there was an interaction effect between different treatment modalities and follow-up time, with a greater decrease in the drug-treated group as follow-up time increased (See Fig. 3).

**Table 2:** Changes in blood uric acid in MDT surgery group and drug group

follow-up time	uric acid(µmol/l)		t	*P*
	MDT Surgical Group	drug group		
Base line	480.00 (195.00)	563.00 (150.50)	3.484	0.001
2 W	427.00 (174.00)	469.00 (175.00)	2.104	0.049
1 M	371.00 (137.00)	382.00 (134.00)	1.338	0.183
3 M	311.00 (58.00)	372.00 (87.75)	5.942	<0.001
6 M	311.00 (57.50)	333.00 (94.00)	1.054	0.294
F	61.53	78.19		
P	<0.001	<0.001		

#### SF-36 scores

**Fig. 4:**
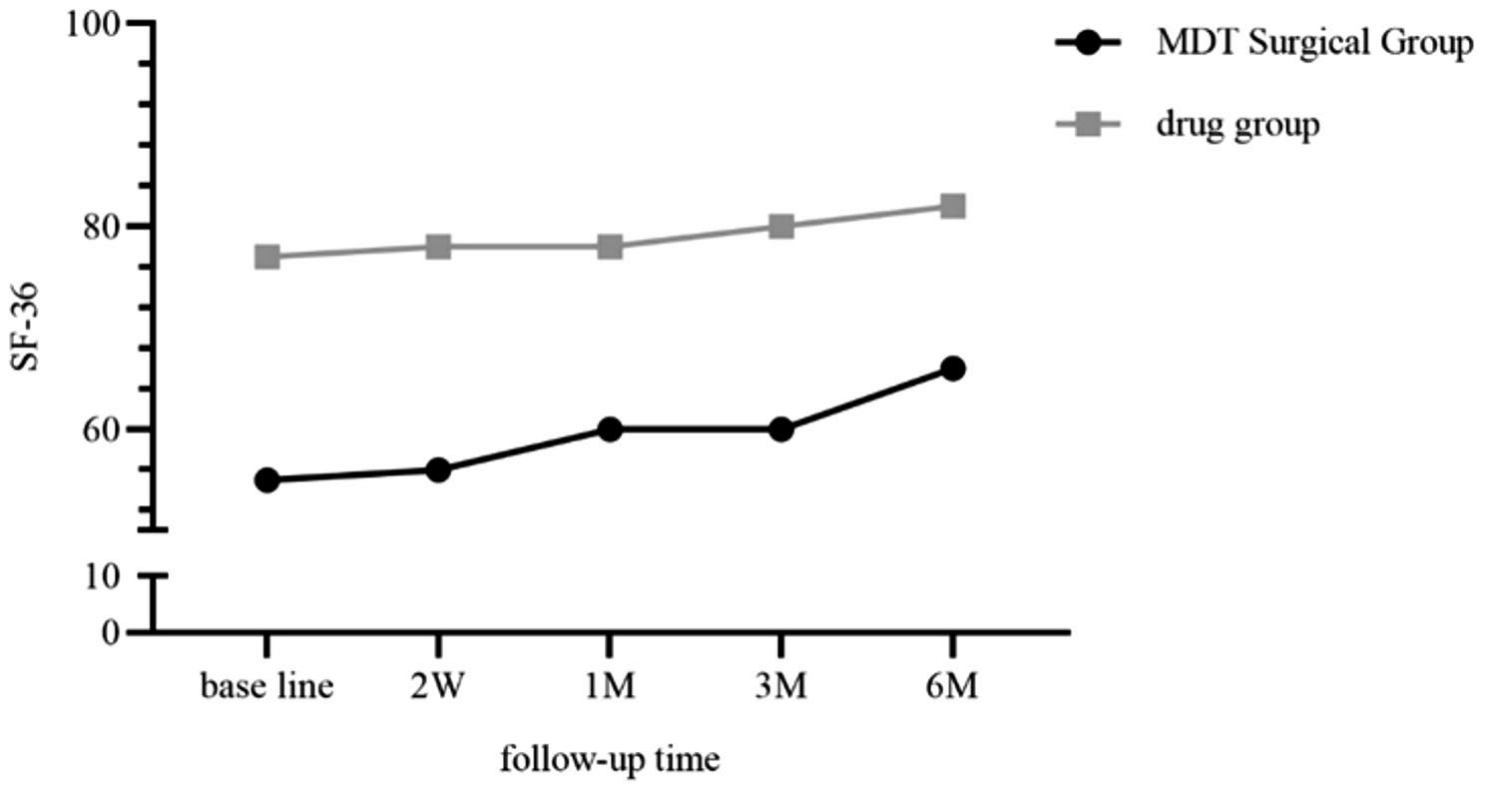
SF-36 over time. (F:36.921, *P*<0.001; F _interaction_:1.405, P _interaction_:0.247; F_intergroup_:72.741, P_intergroup_:<0.001)

There was a statistically significant difference in SF-36 scores before MDT surgery treatment and at multiple follow-ups (F = 29.77, *p* < 0.001). SF-36 scores were significantly higher in January, March, and June after treatment compared to pre-treatment, and the difference was statistically significant (*P* < 0.05) (See Table 3). The difference in SF-36 scores at 6 months after treatment in different modalities was statistically significant, with a greater rise in the MDT surgical treatment group as the follow-up time increased (See Fig. 4).

**Table 3:** Changes in SF-36 in the MDT surgery group and the drug group

follow-up time	SF-36		t	*P*
	MDT Surgical Group	drug group		
Base line	55.00 (24.00)	77.00 (19.50)	10.173	<0.001
2 W	56.00 (20.00)	78.00 (15.00)	0.171	<0.001
1 M	60.00 (20.00)	78.00 (11.50)	0.064	<0.001
3 M	60.00 (20.00)	80.00 (11.50)	0.054	<0.001
6 M	66.00 (19.00)	82.00 (10.50)	0.056	<0.001
F	29.77	16.18		
P	<0.001	<0.001		

### Analysis of the efficacy of MDT surgery and drug treatment on the study population

**Fig. 5:**
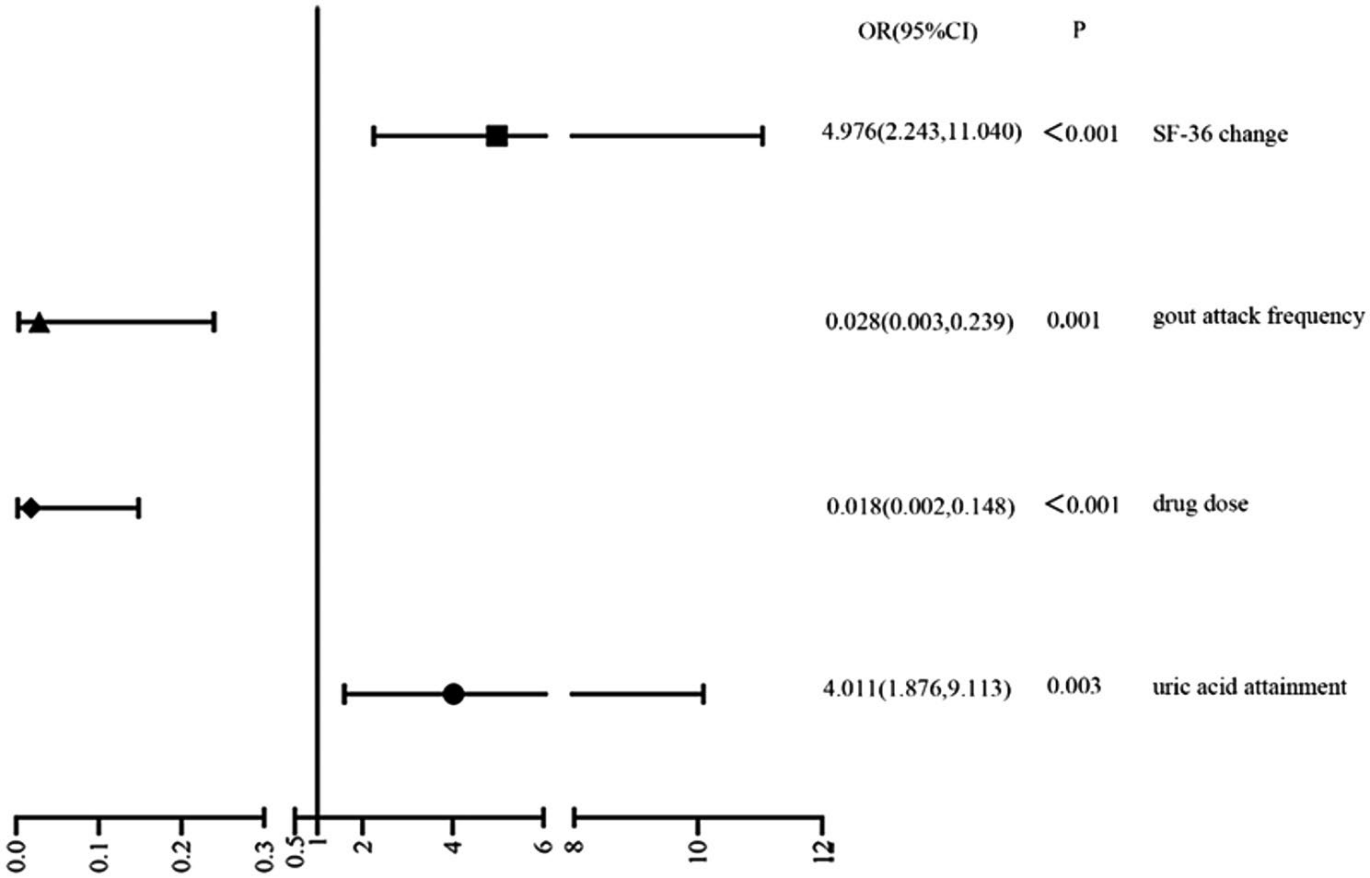
Forest plot of treatment effects after adjusting for confounding variables

Logistic regression analysis was performed with MDT surgery/medication as the independent variable, and whether uric acid reached the standard, satisfaction or not, whether the dose of medication was larger or not, whether gout was frequent or not, whether SF-36 score was better or not, and whether HAQ score was better or not as the dependent variable, respectively, and the results showed that MDT surgical treatment was a protective factor for reaching the standard of uric acid, SF-36 score was better, and it was a protective factor for the use of larger dose of medication, a risk factor for the frequency of gout. After adjusting for gender, age, BMI, smoking history, drinking history, family history, duration of gout, number of gout stone sites, and frequency of gout attacks, the proportion of patients with MDT surgical treatment who reached the uric acid standard was 4.011 times higher than that of medication (OR: 4.011, 95% CI: 1.595, 10.086, *P* = 0.003); the use of higher-dose drugs was 1.8% (OR: 1.8%, 95% CI: 1.595, 10.086, *P* = 0.003) of that of patients with medication. 1.8% (OR: 0.018, 95% CI: 0.002, 0.148, *P* < 0.001); the probability of frequent gout was 2.8% (OR: 0.028, 95% CI: 0.003, 0.2398, *P* = 0.001) of that of drug-treated patients; and the proportion of SF-36 improvement was 4.976 times higher than that of drug treatment (OR: 4.976, the 95% CI: 2.243, 11.040, *P* < 0.001) (See Table 4; Fig. 5).

**Table 4:** Logistic regression analysis of treatment effects

		OR	95%CI	*P*
Uric acid attainment	Model 1	4.134	1.876, 9.113	<0.001
	Model 2	4.196	1.893, 9.300	<0.001
	Model 3	4.011	1.595, 10.086	0.003
Drug dose	Model 1	0.022	0.003, 0.165	<0.001
	Model 2	0.021	0.003, 0.161	<0.001
	Model 3	0.018	0.002, 0.148	<0.001
Gout attack frequency	Model 1	0.034	0.004, 0.256	0.001
	Model 2	0.033	0.004, 0.253	0.001
	Model 3	0.028	0.003, 0.239	0.001
SF-36 change	Model 1	4.941	2.466, 9.900	<0.001
	Model 2	5.154	2.545, 10.437	<0.001
	Model 3	4.976	2.243, 11.040	<0.001

Model 1: unadjusted for variables

Model 2: adjusted for sex, age, and BMI

Model 3: adjusted for smoking history, alcohol consumption history, family history, disease duration, number of gout stone sites, and frequency of gout attacks based on model 2

Uric acid attainment: uric acid < 300 µmol/l defined as 1; drug dose: febuxostat > 40 mg defined as 1; gout attack frequency: gout attack/6 months > 1 defined as 1; SF-36 change: SF-36 (6 M)-SF-36 (baseline) > 0 defined as 1; all referenced to 0

### Effect of MDT surgery and drug treatment on liver function impairment in the study population

In a one-way logistic regression analysis with MDT surgery/medication as the independent variable and the presence of liver function abnormalities as the dependent variable, after adjusting for gender, age, BMI, smoking history, drinking history, family history, duration of gout, number of gout stone sites, and frequency of gouty episodes, MDT surgery was still a protective factor for the presence of liver function abnormalities; compared with the medication group, the presence of liver function abnormalities was 0.317 times higher than that of the drug group (OR: 0.317, 95% CI: 0.121, 0.831, *P* = 0.019) (See Table 5).

**Table 5:** Logistic regression analysis of liver function abnormalities after treatment

	OR	95%CI	*P*
Model 1	0.406	0.178, 0.926	0.032
Model 2	0.394	0.169, 0.919	0.031
Model 3	0.317	0.121, 0.831	0.019

Model 1: unadjusted for variables

Model 2: adjusted for sex, age, and BMI

Model 3: adjusted for smoking history, alcohol consumption history, family history, disease duration, number of gout stone sites, and frequency of gout attacks based on model 2

## Discussion

Gout stones are the main manifestation of advanced gout, and medications are used as the primary treatment for gout, but some patients may have specific genetic mutations or variants that limit the effectiveness of uric acid-lowering medications. A family history of gout, unhealthy lifestyle, differences in medication compliance, comorbidities, and interactions between other types of medications may all make it difficult for patients to achieve the desired uric acid control goals with medication, leading to recurrent flare-ups and the eventual need for surgical intervention. Surgical removal of gout stones can reduce the total amount of uric acid in the body, slow down the progression of the lesion, and prevent irreversible damage to bones, joints, tendons, and nerves [19]. With the help of three-dimensional CT image surgery, the surgical scope can be clearer, and deeply hidden urate crystals can be completely removed to reduce uric acid residue. There are fewer studies on the efficacy of gout stone surgery and the efficacy of gout stone surgery under multidisciplinary integration (MDT). In this study, by comparing patients treated with MDT gout stone surgery, the results show that 3D reconstruction technology multidisciplinary fusion treatment has better efficacy for gout stone patients.

By comparing the baseline data of patients in the MDT surgery group and the drug group, the two groups were comparable. Gout is often combined with diabetes mellitus, hyperlipidemia, kidney stones, coronary artery disease, and hypertension [20]; with the prolonged course of gout, gout stones will gradually appear, involving multiple joints, and the frequency of attacks is elevated in patients with poorly controlled gout.The patients in the MDT surgery group had more co-morbidities and a longer course of gout, with a greater proportion of multi-joint gout stones and more frequent gouty attacks. The baseline blood uric acid was higher in the drug group than in the MDT surgery group in this study, and we hypothesized that this was related to the patients in the MDT surgery group undergoing stricter dietary management and application of medication due to more severe gouty conditions; however, the dietary habits and previous medication history were not investigated and analyzed in the present study, and studies are still needed to further substantiate the reasons for the difference in blood uric acid between the two groups.

During follow-up, uric acid levels decreased and SF-36 scores increased in both groups. After adjusting for confounding factors, multivariate logistic regression analysis showed that the rate of uric acid compliance and the increase of SF-36 were higher in the MDT surgery group. In the MDT group, the proportion of patients receiving high-dose drug therapy, the proportion of patients developing frequent gout, and the proportion of patients with abnormal liver function were lower.

Our study found a gradual decrease in blood uric acid levels over time after patient treatment, with a greater decrease in the drug group of patients. This may be related to the differences in blood uric acid levels at the time of enrollment as well as drug dosage between the two groups of patients. Surgery can remove subcutaneous gout stones and reduce the urate load in the body, but the current study did not find that surgery can directly reduce blood uric acid. The uric acid-lowering drug applied in this study was febuxostat, and previous studies have shown that the uric acid-lowering effect of febuxostat is dose-dependent, and the magnitude of uric acid-lowering is greater as the dose of the drug used increases [21–23]. In patients with comorbid gouty stones, dissolution of urate crystals deposited in the subcutis, tendons, and bones is possible when blood uric acid is < 300 µmol/l [11, 12, 24]. Using the blood uric acid level of < 300 µmol/l at 6 months of follow-up as the criterion for uric acid compliance, and logistic regression with whether or not uric acid compliance was the dependent variable, patients in the MDT surgery group had a higher rate of uric acid compliance, and after adjusting for confounding variables, the rate of uric acid compliance for patients in the MDT surgery group was approximately four times higher than that in the drug group (OR: 4.011). Previous studies also support this finding that patients with gout stones removed after surgical intervention have better control of blood uric acid [19]. During uric acid reduction, urate crystals dissolve and enter the bloodstream, causing an increase in blood uric acid levels; surgery reduces the urate load, resulting in a smooth decrease in blood uric acid during uric acid reduction.

In addition, we followed up the frequency of gouty attacks with medication use. Using more than 40 mg of medication as the delineation criterion and regression analysis after adjusting for confounders, the proportion of patients using larger doses of medication in the MDT surgical group was 1.8% of that in the medication group (OR: 0.018); using more than one gouty attack as the delineation criterion and regression analysis after adjusting for confounders, the proportion of patients experiencing frequent gouty attacks was significantly smaller in the MDT The proportion of patients with frequent gout attacks in the surgery group was significantly smaller than that in the drug group (OR: 0.028). Patients treated with MDT surgery had a lower degree of re-precipitation of uric acid from tissues after blood uric acid decreased than patients without surgery to reduce the urate load. The application of a conventional dose (40 mg once daily) resulted in a smooth decrease in uric acid. Previous studies have found that recurrent gout attacks are associated with gout stones, and serum urate levels correlate with the number of gout stones [25]. The high urate burden associated with gout stones is more likely to lead to recurrent acute gout attacks.

In our study, the collection of SF-36 scores was performed at baseline and follow-up nodes, containing relevant psychosomatic aspects [26]. At the initial quality of life related evaluation, SF-36 scores of patients in the MDT surgery group were lower than those of the drug group, and patients in the MDT surgery group suffered from recurrent episodes of pain, which interfered with activity, sleep, and a decrease in SF-36 scores. The improvement in SF-36 scores was greater in the MDT surgery group during follow-up, and after adjusting for confounders and performing regression analyses, the patients in the MDT surgery group SF-36 score improvement was approximately five times higher than that of the drug group (OR: 4.976), and the results showed that MDT surgery to remove gout stones significantly improved patients’ quality of life.

Hepatic impairment is the most common digestive injury associated with the use of uric acid-lowering drugs (ULTs) [27–32], manifested by an increase in alanine aminotransferase and glutamine aminotransferase [33–35]. The hepatic injury that occurs after the application of febuxostat is considered to be pharmacologic, and the degree of hepatic injury is dose-dependent to some extent. In this study, the effect of different treatment modalities on liver impairment was analyzed after adjusting for confounding variables, and the results showed that patients in the MDT surgery group were 68.3% less likely to develop liver impairment after treatment than those in the drug group (OR: 0.317). The larger proportion of patients in the drug group who used high doses of drugs was one of the reasons for this result.

This study has certain limitations. Firstly, although this retrospective study provides preliminary evidence of the clinical advantages of MDT treatment, prospective studies are required in the future to further validate the robustness and reliability of the results. Additionally, this study primarily compared the preliminary clinical outcomes of MDT surgery with those of traditional surgery, but the lack of multi-center, randomized controlled trials limits the generalizability of the findings. Future studies should include larger sample sizes and multi-center randomized controlled trials to further clarify the efficacy and safety of MDT treatment.

## Electronic supplementary material

Below is the link to the electronic supplementary material.

Supplementary Material 1

Supplementary Material 2

## Data Availability

The data involved in this article has not been uploaded to a public database and can be obtained by contacting the authors.

## References

[1] Dehlin M, Jacobsson L, Roddy E. Global epidemiology of gout: prevalence, incidence, treatment patterns and risk factors. Nat Rev Rheumatol. 2020;16:380–90. 32541923 10.1038/s41584-020-0441-1

[2] Yu KH, Chen DY, Chen JH, Chen SY, Chen SM, Cheng TT, Hsieh SC, Hsieh TY, Hsu PF, Kuo CF, Kuo MC, Lam HC, Lee IT, Liang TH, Lin HY, Lin SC, Tsai WP, Tsay GJ, Wei JC, Yang CH, Tsai WC. Management of gout and hyperuricemia: multidisciplinary consensus in Taiwan. Int J Rheum Dis. 2018;21:772–87. 29363262 10.1111/1756-185X.13266

[3] Ruoff G. N.L. Edwards 2016 Overview of serum uric acid treatment targets in gout: why Less Than 6 mg/dL? Postgrad Med 128 706–15. 27558643 10.1080/00325481.2016.1221732

[4] Sapsford M, Gamble GD, Aati O, Knight J, Horne A, Doyle AJ, Dalbeth N. Relationship of bone erosion with the urate and soft tissue components of the gout stone in gout: a dual energy computed tomography study. Rheumatology (Oxford). 2017;56:129–33. 27803304 10.1093/rheumatology/kew383

[5] Chandratre P, Mallen C, Richardson J, Muller S, Hider S, Rome K, Blagojevic-Bucknall M, Roddy E. Health-related quality of life in gout in primary care: baseline findings from a cohort study. Semin Arthritis Rheum. 2018;48:61–9. 29398125 10.1016/j.semarthrit.2017.12.005 PMC6089841

[6] Stewart S, Dalbeth N, Otter S, Gow P, Kumar S, Rome K. Clinically-evident tophi are associated with reduced muscle force in the foot and ankle in people with gout: a cross-sectional study. J Foot Ankle Res. 2017;10:25. 28649283 10.1186/s13047-017-0207-4 PMC5477352

[7] Zhao X, Tsujimoto T, Kim B, Katayama Y, Tanaka K. Association of Foot structure with the strength of muscles that move the ankle and physical performance. J foot Ankle Surgery: Official Publication Am Coll Foot Ankle Surg. 2018;57:1143–7. 10.1053/j.jfas.2018.06.002 30368426

[8] Dalbeth N, Collis J, Gregory K, Clark B, Robinson E, McQueen FM. Tophaceous joint disease strongly predicts hand function in patients with gout. Rheumatology (Oxford). 2007;46:1804–7. 17982165 10.1093/rheumatology/kem246

[9] Vincent ZL, Gamble G, House M, Knight J, Horne A, Taylor WJ, Dalbeth N. Predictors of mortality in people with recent-onset gout: a prospective observational study. J Rhuematol. 2017;44:368–73. 10.3899/jrheum.160596 27980010

[10] Pagidipati NJ, Clare RM, Keenan RT, Chiswell K, Roe MT, Hess CN. Association of gout with Long-Term Cardiovascular outcomes among patients with obstructive coronary artery disease. J Am Heart Association. 2018;7:e009328. 10.1161/JAHA.118.009328 PMC6201404 30369327

[11] Khanna D, Fitzgerald JD, Khanna PP, Bae S, Singh MK, Neogi T, Pillinger MH, Merill J, Lee S, Prakash S, Kaldas M, Gogia M, Perez-Ruiz F, Taylor W, Lioté F, Choi H, Singh JA, Dalbeth N, Kaplan S, Niyyar V, Jones D, Yarows SA, Roessler B, Kerr G, King C, Levy G, Furst DE, Edwards NL, Mandell B, Schumacher HR, Robbins M, Wenger N, Terkeltaub R. 2012 American College of Rheumatology guidelines for management of gout. Part 1: systematic nonpharmacologic and pharmacologic therapeutic approaches to hyperuricemia. Arthritis Care Res. 2012;64:1431–46. 10.1002/acr.21772 PMC3683400 23024028

[12] Perez-Ruiz F, Martin I, Canteli B. Ultrasonographic measurement of tophi as an outcome measure for chronic gout. J Rhuematol. 2007;34:1888–93. 17659752

[13] Peiteado D, Villalba A, Martín-Mola E, Balsa A, De Miguel E. Ultrasound sensitivity to changes in gout: a longitudinal study after two years of treatment. Clin Exp Rheumatol. 2017;35:746–51. 28281462

[14] Yang DH, Chen HC, Wei JC. Early urate-lowering therapy in gouty arthritis with acute flares: a double-blind placebo controlled clinical trial. Eur J Med Res. 2023;28:10. 36609359 10.1186/s40001-022-00982-8 PMC9817311

[15] Hu AM, Brown JN. Comparative effect of allopurinol and febuxostat on long-term renal outcomes in patients with hyperuricemia and chronic kidney disease: a systematic review. Clin Rheumatol. 2020;39:3287–94. 32418037 10.1007/s10067-020-05079-3

[16] Li S, Xu G, Liang J, Wan L, Cao H, Lin J. The role of Advanced imaging in gout management. Front Immunol. 2021;12:811323. 35095904 10.3389/fimmu.2021.811323 PMC8795510

[17] Schwabl C, Taljanovic M, Widmann G, Teh J, Klauser AS. Ultrasonography and dual-energy computed tomography: impact for the detection of gouty deposits. Ultrasonography (Seoul Korea). 2021;40:197–206. 33307617 10.14366/usg.20063 PMC7994744

[18] Neogi T, Jansen TL, Dalbeth N, Fransen J, Schumacher HR, Berendsen D, Brown M, Choi H, Edwards NL, Janssens HJ, Lioté F, Naden RP, Nuki G, Ogdie A, Perez-Ruiz F, Saag K, Singh JA, Sundy JS, Tausche AK, Vaquez-Mellado J, Yarows SA, Taylor WJ. 2015 gout classification criteria: an American College of Rheumatology/European League against Rheumatism collaborative initiative. Ann Rheum Dis. 2015;74:1789–98. 26359487 10.1136/annrheumdis-2015-208237 PMC4602275

[19] Lee SS, Chen MC, Chou YH, Lin SD, Lai CS, Chen YC. Timing of intra-lesion shaving for surgical treatment of chronic gout stone. J Plast Reconstr Aesthetic Surgery: JPRAS. 2013;66:1131–7. 23702196 10.1016/j.bjps.2013.03.041

[20] Singh JA, Gaffo A. Gout epidemiology and comorbidities. Semin Arthritis Rheum. 2020;50:S11–6. 32620196 10.1016/j.semarthrit.2020.04.008

[21] Zhang F, Liu Z, Jiang L, Zhang H, Zhao D, Li Y, Zou H, Wang X, Li X, Shi B, Xu J, Yang H, Hu S, Qu S, Randomized A. Double-Blind, Non-inferiority Study of Febuxostat Versus Allopurinol in Hyperuricemic Chinese subjects with or without gout. Rheumatol Therapy. 2019;6:543–57. 10.1007/s40744-019-00173-8 PMC6858416 31531831

[22] Hosoya T, Furuno K, Kanda S. A non-inferiority study of the novel selective urate reabsorption inhibitor dotinurad versus febuxostat in hyperuricemic patients with or without gout. Clin Exp Nephrol. 2020;24:71–9. 31970593 10.1007/s10157-020-01851-6 PMC7066279

[23] Jordan A, Gresser U. Side effects and interactions of the Xanthine oxidase inhibitor febuxostat. Pharmaceuticals (Basel Switzerland). 2018;11(2):51. 10.3390/ph11020051 PMC6027216 29799494

[24] Perez-Ruiz F, Calabozo M, Pijoan JI, Herrero-Beites AM, Ruibal A. Effect of urate-lowering therapy on the velocity of size reduction of tophi in chronic gout. Arthritis Rheum. 2002;47:356–60. 12209479 10.1002/art.10511

[25] Khanna PP, Nuki G, Bardin T, Tausche AK, Forsythe A, Goren A, Vietri J, Khanna D. Tophi and frequent gout flares are associated with impairments to quality of life, productivity, and increased healthcare resource use: results from a cross-sectional survey. Health Qual Life Outcomes. 2012;10:117. 22999027 10.1186/1477-7525-10-117 PMC3499162

[26] Alzahrani O, Fletcher JP, Hitos K. Quality of life and mental health measurements among patients with type 2 diabetes mellitus: a systematic review. Health Qual Life Outcomes. 2023;21:27. 36949507 10.1186/s12955-023-02111-3 PMC10031182

[27] Oh YJ, Moon KW. Combined use of Febuxostat and Colchicine does not increase Acute Hepatotoxicity in patients with gout: a retrospective study. J Clin Med. 2020;9(5):1488. 10.3390/jcm9051488 PMC7290683 32429082

[28] Ishikawa T, Takahashi T, Taniguchi T, Hosoya T. Dotinurad: a novel selective urate reabsorption inhibitor for the treatment of hyperuricemia and gout. Expert Opin Pharmacother. 2021;22:1397–406. 33926357 10.1080/14656566.2021.1918102

[29] Wakabayashi T, Ueno S, Nakatsuji T, Hirai T, Niinomi I, Oyama S, Inada A, Kambara H, Iwanaga K, Hosohata K. Safety profiles of new xanthine oxidase inhibitors: a post-marketing study. Int J Clin Pharmacol Ther. 2021;59:372–7. 33560211 10.5414/CP203898

[30] Liang N, Sun M, Sun R, Xu T, Cui L, Wang C, Ma L, Cheng X, Xue X, Sun W, Yuan X, Zhang H, Li H, He Y, Ji A, Wu X, Li C. Baseline urate level and renal function predict outcomes of urate-lowering therapy using low doses of febuxostat and benzbromarone: a prospective, randomized controlled study in a Chinese primary gout cohort. Arthritis Res Therapy. 2019;21:200. 10.1186/s13075-019-1976-x PMC6719374 31477161

[31] Gunawardhana L, Becker MA, Whelton A, Hunt B, Castillo M, Saag K. Efficacy and safety of febuxostat extended release and immediate release in patients with gout and moderate renal impairment: phase II placebo-controlled study. Arthritis Res Therapy. 2018;20:99. 10.1186/s13075-018-1593-0 PMC5977466 29848361

[32] Lee JS, Won J, Kwon OC, Lee SS, Oh JS, Kim YG, Lee CK, Yoo B, Hong S. Hepatic Safety of Febuxostat Compared with allopurinol in gout patients with fatty liver disease. J Rhuematol. 2019;46:527–31. 10.3899/jrheum.180761 30442825

[33] Hosoya T, Sasaki T, Hashimoto H, Sakamoto R, Ohashi T. Clinical efficacy and safety of topiroxostat in Japanese male hyperuricemic patients with or without gout: an exploratory, phase 2a, multicentre, randomized, double-blind, placebo-controlled study. J Clin Pharm Ther. 2016;41:298–305. 27079434 10.1111/jcpt.12392

[34] Dalbeth N, Saag KG, Palmer WE, Choi HK, Hunt B, MacDonald PA, Thienel U, Gunawardhana L. Effects of Febuxostat in Early Gout: A Randomized, Double-Blind, Placebo-Controlled Study. Arthritis & rheumatology (Hoboken, N.J.). 2017;69:2386–2395. 10.1002/art.40233 PMC5725733 28975718

[35] Jalal DI, Decker E, Perrenoud L, Nowak KL, Bispham N, Mehta T, Smits G, You Z, Seals D, Chonchol M, Johnson RJ. Vascular function and uric acid-lowering in Stage 3 CKD. J Am Soc Nephrology: JASN. 2017;28:943–52. 10.1681/ASN.2016050521 PMC5328166 27620990

