# Supplementary material for: Analysis of the efficacy of multidisciplinary integration based on 3D reconstruction technology for the treatment of gout stone
Source: J Orthop Surg Res. 2025 Feb 4;20:132. doi: 10.1186/s13018-025-05506-8 (PMC11792198; doi:10.1186/s13018-025-05506-8)
Supplement: Supplementary file 1 — Supplementary Material 1 [file 13018_2025_5506_MOESM1_ESM.pdf]

85 patients in the MDT surgery group and 57 patients in the conventional surgery group were included in the study.The differences between the two groups in terms of gender, age, height, weight, BMI, systolic blood pressure, diastolic blood pressure, history of smoking, history of alcohol consumption, duration of gout, ghrelin, ghrelin transaminase, ghrelin transaminase, urea nitrogen, creatinine, blood glucose, triglycerides, and total cholesterol levels were not statistically significant (P > 0.05). The differences in the MDT surgery group between the patients with a family history of gout, multiple joint gout stones, and frequent gout accounted for more, and the difference was not statistically significant (P > 0.05). (See Supplementary Table 1)

Supplementary Table 1 Comparison of general data of gouty stone patients with or without MDT surgery

Characteristics	MDT Surgical Group（n=85）	Traditional Surgery Group（n=57）	P
Males,n(%)	83 (97.65%)	56 (98.25%)	0.917
Age(years)	50.85±6.43	51.14±5.31	0.935
BMI (kg/m2)	26.85±4.80	26.71±4.13	0.825
SBP(mmHg)	142.75±20.74	143.66±21.17	0.867
DBP(mmHg)	86.40±13.97	87.35±12.47	0.772
Smoking	40 (47.06%)	32 (56.14%)	0.172
Drinking	48 (56.47%)	37 (64.91%)	0.260
Family history	18 (21.18%)	11 (19.29%)	0.673
Course of gout(years)	10.00 (11.00)	10.00 (10.00)	0.803
Multiple tophus,n(%)	73 (85.88%)	48 (84.21%)	0.881
Frequent gout attacks,n(%)	78 (91.76%)	50 (87.72%)	0.543
ALT	23.00 (19.00)	24.00 (19.50)	0.793
AST	20.00 (10.00)	21.00 (9.50)	0.952
GLU	5.10±1.42	5.40±1.30	0.877
TG	1.56 (0.95)	1.68 (0.85)	0.228
TC	4.55±0.95	4.85±1.03	0.653
BUN	6.10 (3.02)	5.20 (3.05)	0.210
CREA	80.00 (26.00)	89.00 (21.5)	0.133

There was no statistically significant difference between the preoperative blood uric acid levels of the two groups; the blood uric acid levels of the two groups decreased at 6 months postoperatively compared with the preoperative levels, and compared with the traditional surgery group, the blood uric acid levels of the patients in the MDT surgery group were lower at 6 months postoperatively, and the difference was statistically significant (P < 0.001). (See Supplementary Table 2)

Supplementary Table 2 Changes in blood uric acid levels in gouty stone patients with or without MDT surgery

Group	N	base line	6M
MDT Surgical Group	85	480.00±51.00	311.00±57.50
Traditional Surgery Group	57	467.00±33.00	366.00±47.00
P		0.661	＜0.001

The above suggests that MDT patients gained additional clinical benefit in terms of postoperative uric acid control in gout patients compared with conventional surgery.
